# Red Blood Cells: A Newly Described Partner in Central Retinal Vein Occlusion Pathophysiology?

**DOI:** 10.3390/ijms24021072

**Published:** 2023-01-05

**Authors:** Sandrine Laurance, Mickaël Marin, Yves Colin

**Affiliations:** Université Paris Cité and Université des Antilles, INSERM, BIGR, F-75014 Paris, France

**Keywords:** red blood cell, vascular occlusion, erythropoiesis, adhesion, visual loss

## Abstract

Central retinal vein occlusion (CRVO) is a frequent retinal disorder inducing blindness due to the occlusion of the central vein of the retina. The primary cause of the occlusion remains to be identified leading to the lack of treatment. To date, current treatments mainly target the complications of the disease and do not target the primary dysfunctions. CRVO pathophysiology seems to be a multifactorial disorder; several studies did attempt to decipher the cellular and molecular mechanisms underlying the vessel obstruction, but no consensual mechanism has been found. The aim of the current review is to give an overview of CRVO pathophysiology and more precisely the role of the erythroid lineage. The review presents emerging data on red blood cell (RBC) functions besides their role as an oxygen transporter and how disturbance of RBC function could impact the whole vascular system. We also aim to gather new evidence of RBC involvement in CRVO occurrence.

## 1. Introduction

Central retinal vein occlusion (CRVO) is an elderly affecting disorder leading to visual loss. Impairment of visual acuity is the result of the obstruction of the blood flow of the retinal venous system. Despite the high prevalence of this disabling condition, the underlying pathophysiology is far from being elucidated and seems to be the consequence of dysfunctions of blood circulating and/or endothelial cells (ECs). The vasculature is a complex system that comprises ECs, which are highly specialized cells involved in a wide range of physiological and pathological processes but also blood circulating cells. Endothelial cells form a single cell layer at the interface between blood flow and the surrounding tissue. Talking about ECs is imprecise as endothelial cells gather several subtypes depending on their localization. In fact, ECs have a different phenotype depending on if they belong to the micro- or macro-vasculature and also the microenvironment of the organ or tissue they irrigate. They are involved in a wide range of physiological and pathological processes such as the regulation of oxygen and nutrient supplies to tissue, coagulation, and inflammation. They are in close contact with blood-circulating cells. These latter include red blood cells (RBC), leukocytes, and platelets whose functions range from oxygen-carrying to immunity and coagulation. Interactions and communications between ECs and blood-circulating cells are complex and reciprocal. Any disturbance in ECs or blood circulating cells functioning will definitively impact the functioning of their counter-partners. The role of ECs in vascular disorders has been extensively studied as well as the role of leukocytes or platelets whereas RBCs were considered for a long time as inert bystanders. However, since the last decade RBCs have moved from a passive to an active role in these disorders notably cardiovascular disorders.

The aim of this review is to give an overview of CRVO pathogenesis and to compile novel evidence on the role of RBCs in several disorders and more specifically how RBC dysfunctions would be involved in CRVO occurrence.

## 2. Central Retinal Vein Occlusion

CRVO is the second most frequent retinal vascular disorder after diabetic retinopathy with an estimated age- and sex-standardized prevalence of 0.8 per 1000 [1]. Epidemiological studies evaluate that around 4.67 million people are living with CRVO worldwide with most of the patients between 70 and 79 years old [2,3]. CRVO is characterized by the occlusion of the venous network of the retina near or at the lamina cribosa of the optic nerve, ultimately resulting in visual loss [2,4]. To date, clinical studies have failed to identify specific risk factors. The major identified risk factors are increasing age, coexisting cardiovascular disorders, systemic hypertension, and glaucoma [5,6,7,8]. These risk factors are not specific to CRVO and have also been identified as risk factors for other elderly disorders such as diabetes, hypertension, and cardiovascular complications.

CRVO’s common clinical complications range from macular edema, macular ischemia, retinal hemorrhages, and venous tortuosity and dilation [9]. Neovascular glaucoma is one of the worst complications as it considerably increases the risk of blindness [10]. One of the main consequences of the occlusion of the central retinal vein is the formation of an ischemic, hypoxic, and pro-inflammatory milieu leading to macula edema and retinal neovascularization due in part to endothelial cell activation. However, the leading causes of the vessel obstruction remain not clearly identified and several mechanisms have been explored such as thrombus formation, hypercoagulability, or platelet dysfunction. One of the main reasons for this absence of a detailed pathogenesis of CRVO is the variability in the clinical presentation [11]. As described by Hayreh, CRVO can be classified in ischemic and non-ischemic forms [12]. Ischemic CRVO occurs in 20 to 35% of CRVO patients and is characterized by huge retinal hemorrhages and wide areas of non-perfusion. These patients are prone to develop neovascularizations and glaucoma, and usually exhibit a severe loss of vision. Non-ischemic CRVO develops in 75% of CRVO patients who exhibit no retinal non-perfusion and a good vision at CRVO presentation and are prone to maintain a good vision in the long term. Fifteen to thirty percent of the non-ischemic form can progress to an ischemic form thus worsening the visual prognosis.

## 3. Central Retinal Vein Occlusion: Not a Simple Thrombotic Disorder

If the post-occlusion events are well described and explored, the initial cause of the occlusion remains to be elucidated and is still under debate. One of the most obvious hypotheses for CRVO occurrence is that the occlusion would be consecutive to the formation of a thrombus in the central retinal vein. Vessel thrombus formation is classically described as the consequence of dysregulation of one of the elements of the famous Virchow’s triad [13]. The latter includes three parameters: vessel wall integrity, coagulation state, and blood flow. Any disturbance of any of these parameters is susceptible to inducing venous thrombosis. Several clues on CRVO pathogenesis led us to think that the observed occlusion may result from clot formation. Systemic vascular comorbidities such as hypertension, diabetes, and obesity atherosclerosis can lead to the activation of endothelial cells of the central retinal vein wall and thus induce a pro-thrombotic state by modifying the blood pressure and blood hemodynamics [14,15]. However, the underlying mechanism involved in CRVO pro-thrombotic propensity remains to be fully described. Thrombus formation is a tightly regulated physiological process involving complex interactions between endothelial cells, exposed subendothelial matrix, platelets, and fibrin. After thrombus formation, the anti-coagulation system is activated and will stop clot formation. Any disturbance of any mediators of the pro- or anti-coagulation systems would lead to an increased risk of thrombosis [16]. In CRVO patients, fluorescent angiography shows a slowdown of the blood flow rather than a complete obstruction of the vein which strongly suggests the absence of thrombus [17]. Some anatomopathological studies were performed showing that the presence of a thrombus is not consistent [18].

Several studies have investigated the role of platelets as well as thrombotic factors in CRVO occurrence. To date, the conclusions of these studies are still under debate and the biological relevance of any mutation or dysfunction of the hemostatic system in CRVO remains to be clearly stated. An association between factor V Leiden, protein C, protein S, antithrombin III, and factor XII in CRVO pathobiology has been found in some studies but several others failed to report any association [19,20,21,22]. As reviewed by Marcinkowka et al., these discordances could be explained by the different methodologies applied in the several studies and many limitations have been identified such as appropriate control population or sample size [23,24].

Platelets are known to play an active role in thrombo-occlusive events. Mean platelet volume (MPV) is the main marker of platelet activation [25]. As for coagulation mediators, results regarding platelet involvement in CRVO occurrence are still under debate. Several studies have reported an association between MPV and CRVO [26,27], thus reinforcing studies showing that platelet aggregation is increased in CRVO [28]. Hayreh et al. reported that several hematological abnormalities, not only MPV, can be associated with CRVO occurrence but with high variability [29].

To date, no reliable association between platelet or coagulability markers and CRVO pathobiology has been proven meaning that the source of occlusion is not consecutive to clot formation per se. This observation is reinforced by the fact that anti-thrombotic treatments did show inconsistent efficacy [30]. The lack of an anti-thrombotic drug effect in resolving retinal occlusion highlights the complexity of CRVO pathogenesis which appears to not be a “simple” thrombotic disorder but a multifactorial disorder.

## 4. Central Retinal Vein Occlusion: How to Treat?

Due to an absence of a precise mechanism explaining the primary occurrence of the occlusion, no efficient therapy is available to prevent or cure this disorder meaning that current therapeutical strategies are targeting the complications of this visual disorder but not the source [31]. As the main complications of CRVO result from neovascularization and macular edema, treatments were developed to reduce or block these phenomena. Vascular endothelial growth factor-A (VEGF-A) is a cytokine that promotes notably vascular permeability, and it has been shown that its expression is upregulated in CRVO [32,33,34]. Several anti-VEGF-A therapies were developed either using a monoclonal antibody fragment targeting VEGF-A [35,36,37] or a recombinant protein containing the VEGF-binding domain of the VEGF-receptor 1 and 2 fused to an Fc domain [38,39,40]. Nowadays, about 10 different drugs targeting the VEGF pathway have been designed. Among them, ranibizumab and aflibercept are the two anti-VEGF molecules that were the most studied in randomized clinical trials. Their efficacy and safety have been clearly proven in reducing neovascularization and macular edema. The major disadvantage of these treatments is that they are not permanent and that multiple injections are required. In addition, they do not target the primary cause of the blood flow slowing down, thus they do not prevent any recurrence of the occlusion [41].

Another way to target vascular permeability and macular edema is the use of corticosteroids that would contribute to reducing local inflammation. Either intravitreal triamcinolone acetonide or implantable intravitreal dexamethasone was administrated to CRVO patients and exhibited a strong efficiency to reduce macular edema and inflammation. The major advantage of these treatments is their longer durability (up to 4 months) compared to anti-VEGF therapies. Visual acuity was improved in some patients, but side effects were observed such as an increase in intraocular pressure and an increased risk of cataracts. Consequently, those drugs are infrequently used or used in combination with anti-VEGF treatment [42,43].

Some non-pharmaceutical approaches have been tried such as laser photocoagulation. The latter was the first attempt at CRVO treatment before the advent of corticosteroids and anti-VEGF drugs. Several studies were conducted in the 1970s but none of them manage to prove the efficiency and the safety of this technic [44,45]. A couple of surgical procedures were also investigated but none of these approaches has been investigated in a randomized study and has been proven to be clearly efficient.

There are still some lacks in CRVO pathological process that have to be fulfilled in a way to increase the therapeutic armamentarium.

## 5. Red Blood Cell Dysfunctions and Erythropathy

Red blood cells (RBCs) are the most abundant cell type in the body. Their main function is to transport respiratory gases (oxygen, carbon dioxide, and nitric oxide) throughout tissues; this transporting/circulating feature they are in direct or indirect contact with almost all the different cell types of the organism. For a long time, RBCs were considered only as oxygen bags. For a couple of decades, many studies have highlighted more complex functions for RBCs meaning that they have essential physiological functions beyond being an oxygen carrier. Thus, any disturbance in RBC function would deeply impact the physiology of the vasculature. RBC dysfunctions have been extensively studied in erythroid diseases such as hemoglobinopathies (thalassemia and sickle cell disease). Recent studies showed that alterations in RBC function will result in various dysfunctional phenotypes. These dysfunctions of RBC functions were recently defined as “erythropathy” and can lead to vascular and cardiovascular impairment [46]. This new concept highlights the role of RBC beyond erythroid disorders. RBCs appeared to be key regulators of vascular homeostasis. Until very recently, ECs were presumed to be the principal cellular actor in vascular disorders. Emerging and very compelling data are showing that a primary dysfunction of RBC would in turn trigger EC activation/dysregulation causing vascular imbalance, ultimately resulting in vascular disorder.

Recent findings have clearly established RBC involvement in cardiovascular complications in type 2 diabetes [46], and venous and arterial thrombosis [47]. As reviewed by Weisel et al., they are key players in hemorheological properties, platelets activity, endothelial cell activation, thrombin generation, and clot formation [48]. One of the main properties of RBCs is their deformability. They can adapt their shape to the changing flow and shear stress to facilitate their passage through small capillaries and thus minimize the resistance to the flow. From a discoid shape, the RBC can adapt very quickly its morphology to the local environment. This plasticity is mediated through the high remodeling capacity of the cytoskeleton, specific interactions between the cytoskeleton and plasma membrane, and composition of the plasma membrane and the cytosol (hydration, viscosity, …) [49]. The main actors of this deformability are spectrins, band 3, and the lipid composition of the plasma membrane [50,51]. Any changes in this remarkable flexibility would impair in turn blood hemorheology. Loss of deformability could lead to increases in hematocrit and blood viscosity, RBC aggregation, and small vessel occlusion. RBC deformability property is also linked to the adhesion capacity of the RBC. In physiological conditions, adhesion events between circulating RBCs, endothelial cells, and other circulating cells are negligible. However, in pathological conditions, RBCs become sticky and can activate other circulating cells and ECs. By establishing interactions between the different cell types, RBCs could contribute to vessel occlusion. Depending on the pathological stimulus, the mechanisms of vascular occlusion are different. In the case of deep vein thrombosis or atherosclerosis, the occlusion is the direct consequence of clot formation involving the activation of the coagulation cascade, platelet activation, and fibrin formation [52]. In other disorders such as sickle cell disease or diabetes a slowing down of the blood flow results in an increase in adhesion events [53]. In such disorders, a large panel of adhesion molecules is expressed at the RBC membrane that will mediate the interaction between RBCs and other circulating cells (leukocytes, neutrophils, platelets, ...) but also with ECs. Selectin receptors expressed on RBCs actively participate in leukocytes “rolling” on endothelial cells meaning that RBCs are also involved in inflammation. Other RBC adhesion molecules are involved in firm interactions such as ICAM-4, CD44, and BCAM/Lu [54,55]. The involvement of these adhesion molecules depends on the stimulus leading to the increased adhesion meaning the pathological context. Increased RBC adhesivity has been observed in several disorders in which a vascular obstruction not consecutive to a clot formation is occurring such as SCD, diabetes, and polycythemia vera. For a decade, several studies have also highlighted RBC dysfunction in Gaucher disease (GD). This latter is due to β-glucocerebrosidase (GluCerase) deficiency and is the most common autosomal recessive lysosomal storage disorder. RBCs from Gaucher disorder exhibit higher adhesion properties, abnormal hemorheological properties, and an abnormal shape [56,57,58].

Finally, RBCs can also act on the vascular tone through the release of vasomediators, nitric oxide (NO), S-nitrosothiol (SNO), and adenosine triphosphate (ATP) release [51]. RBCs participate in NO bioavailability, export of NO and ATP, and redox balance [59,60].

Thus, alterations in RBC functions can trigger vascular tone changes, redox imbalance, inflammation through the adhesion process and loss of deformability, and even thrombosis.

## 6. RBC Dysfunctions in CRVO

As much evidence converges to prove that CRVO is not strictly due to the formation of a thrombus and due to the growing evidence highlighting RBCs as key regulators of the vascular system, several studies aimed to investigate the role of RBCs in CRVO occurrence and if impairment in RBC physiology could participate to CRVO pathobiology.

Several studies have investigated the relevance of blood viscosity abnormalities in CRVO occurrence. In fact, an increase in blood viscosity could disturb the blood flow and thus promote the occlusion of the central vein of the retina. It has been shown that several parameters of blood viscosity are altered in CRVO patients such as higher hematocrit, reduced RBC deformability, and enhanced index of erythrocytes aggregation [61,62,63]. It has also been shown that ROS production and lipid peroxidation is increased in erythrocytes from patients suffering from retinal vein occlusion and this would contribute to the observed alteration of hemorheological parameters [64]. Some studies even described the beneficial effects of hemodilution therapy to treat CRVO patients as treated CRVO patients exhibit improvement in visual acuity [65,66,67]. These data reinforce the concept of CRVO as a consequence of disturbance of rheological factors and thus erythroid properties. However, defects in hemorheology are not sufficient to explain the occlusion phenomena of the central vein retina and hemodilution therapy has shown limitation [68].

As described above, RBC biology is complex. Thus, other erythroid parameters besides rheology have been investigated in CRVO occurrence. Interestingly, it has been previously shown that some myeloproliferative disorders such as polycythemia vera (PV) and essential thrombocythemia can induce a large panel of arterial and venous vascular diseases [69,70]. Myeloproliferative disorders are due to a dysregulation in the production of blood cells in the bone marrow usually leading to an unexplained increase in blood cell count. Human erythropoiesis is a complex multi-step process that takes place in the bone marrow in adults, starting from a multipotent hematopoietic stem cell and ending with an enucleated reticulocyte that matures in the circulation to become a red cell [71]. The process of erythropoiesis is known to be mainly regulated by the humoral cytokine erythropoietin (Epo).

One of the major tools to diagnose a latent myeloproliferative disorder is endogenous erythroid formation (EEC) in a cytokine-free medium meaning that erythroid differentiation is occurring in the absence of Epo [72]. Interestingly, Héron et al. described the formation of EEC in 30% of patients presenting an occlusion of the retinal vein [73]. This observation strongly suggests an association between CRVO occurrence and hematological disorders. However, no other clinical, biological, or genetic evidence support this association. Moreover, the V617F mutation JAK2 (an intermediate of Epo signaling), a well-described mutation found in most PV patients, was not found in CRVO patients [73,74,75]. Thus, the mechanism underlying this spontaneous growth of erythroid progenitors in CRVO remains to be fully explored as well as the precise functional consequence.

**Figure 1:**
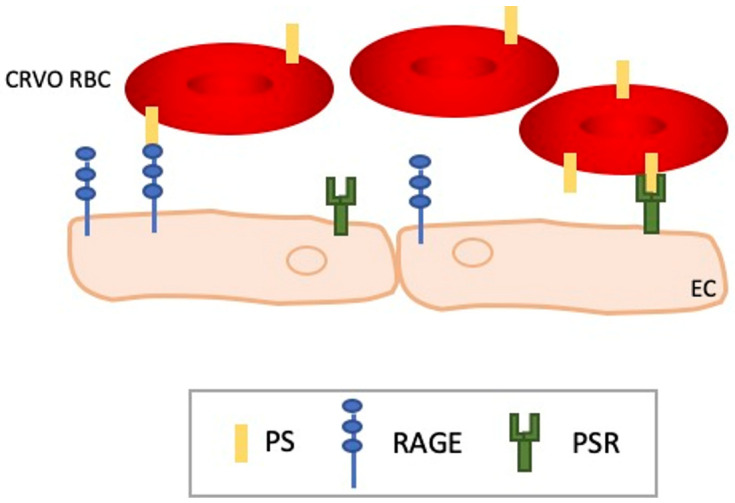
Role of phosphatidylserine exposure in CRVO pathophysiology. PS exposure on CRVO RBCs leads to increase adhesion to endothelial cells through the interaction with PSR and/or RAGE. CRVO RBC adherence to the endothelium contributes to the slowdown of blood flow leading to occlusion.

The physiology of the mature and circulating RBCs seems also to be affected in CRVO patients. Indeed, Wautier et al. did show that RBCs from CRVO patients are more prone to adhere to ECs compared to healthy donors, both in static and flow conditions, but at a lesser extent compared to diabetic or PV patients [76]. Among the panel of analyzed adhesion molecules, only phosphatidylserine (PS) was increased at the surface of CRVO RBC, and this increase was correlated with the increased adhesion. Interestingly, this alteration of CRVO RBC appeared to be permanent as increased adhesion and PS exposure are steady from the time of diagnosis and up to 2 years after the diagnosis. PS is a phospholipid usually located in the inner leaflet of the plasma membrane. Upon cell activation or apoptosis, PS is externalized to the outer leaflet. PS can act as an adhesion molecule and several potential receptors have been identified such as CD36, PS receptor protein, or Stabilin-2 or RAGE, the receptor for advanced glycation end products [77] (Figure 1). In addition to its adhesive function, PS can also promote activation of the coagulation cascade through interaction with factor V and factor VIII. Su et al. did explore PS-exposing cells, microparticles, the coagulation time, purified coagulation complex, and fibrin production [78]. In accordance with the study of Wautier et al., they showed that CRVO patients present a higher rate of PS-exposing erythrocytes but also of platelets and neutrophils. They also showed that those patients exhibit a high rate of circulating microparticles. The latter are small membrane vesicles, less than 1 μm in diameter, released when cells are activated or apoptotic. They are presumed to be involved in several disorders such as thrombosis, inflammation, angiogenesis, or apoptosis. They can arise from ECs, RBCs, leukocytes, or platelets. Su et al. showed that CRVO patients present high levels of MPs originating mainly from platelets followed by RBCs and leukocytes. According to the authors, PS exposure and MPs release would induce impairment in fibrin formation and the coagulation time and these disturbances would greatly contribute to the occlusion of the central retinal vein.

Thus, several erythroid dysfunctions have been identified in CRVO patients (Table 1). However, the molecular mechanisms are still not elucidated as well as the precise involvement in these RBC abnormalities in CRVO pathophysiology. All the affected functions are closely related and any imbalance in one RBC property would lead to another dysfunction.

## 7. Discussion

CRVO is a debilitating disorder affecting the elderly resulting in loss of vision. Despite its prevalence and its health burden, the mechanism leading to this visual impairment remains non-elucidated. As a consequence, few treatments are available, and they mainly target the complications of the disease rather than the cause. In fact, this latter remains unclear and the leading event driving the occlusion is still under investigation. The vessel occlusion was not due to the formation of a thrombus and the pathophysiology is more complicated than a simple dichotomy between thrombotic and non-thrombotic disorders. Thus, CRVO pathophysiology is complex, and it is appeared to be an intricate interplay between endothelial cells and circulating cells. Among them, RBCs have emerged as key players in several vascular disorders.

Despite being enucleated and organelle-free, RBCs are involved in a wide spectrum of physiological functions and any impairment of their physiology could trigger the development of “erythropathy” that could lead to or mediate cardiovascular injuries in several pathological contexts such as type 2 diabetes. These recent observations highlight the crucial role of RBC beyond being an oxygen transporter. Thus, RBCs have recently emerged as active players in vascular disorders and in CRVO. Several erythroid dysfunctions have been identified in CRVO patients such as increased blood viscosity, adhesion, and impairment in erythroid differentiation. However, the molecular mechanism leading to these erythroid dysfunctions is still not identified as well as their exact consequences in CRVO pathobiology. It is crucial to understand more precisely the mechanisms underlying the increase in adhesion, the Epo-independent erythropoiesis, and the hemorheological abnormalities. Several limitations could be observed in the published studies on the role of RBC in CRVO pathophysiology. The sizes of the cohorts are usually small, and the studies often did not discriminate between the different form of retinal vein occlusion. Nevertheless, RBC involvement in CRVO pathobiology is undeniable. Thus, it is crucial to design multi-center research to investigate in randomized cohorts the erythroid biomarkers, functions, morphology, deformability, and production. Several axes should be carefully explored such as adhesion events, PS externalization that could be at the interface between adhesion and coagulation, hemorheology, deformability but also the unexplored abnormal erythroid differentiation. Establishing the link between these dysfunctions and CRVO severity would bring new clues to CRVO patient follow-up.

Deciphering the cause of erythroid dysfunctions would bring new evidence in CRVO pathophysiology but more essentially would provide new potential therapeutic targets.

## Figures and Tables

**Table 1:** Main hypothesis on CRVO occurrence.

Reference	CRVO Occurrence Hypothesis
Kang et al., 2013 [14]	Thrombus formation
Janssen et al., 2005 [15]	
Rehak et al., 2010 [19]	
Williamson et al., 1996 [20]	
Greven et al., 1997 [21]	
Bertram et al., 1995 [22]	
Paques et al., 2005 [17]	Blood flow slow down
Green et al., 1981 [18]	
Park et al., 2002 [25]	Platelet dysfunctions
Pinna et al., 2021 [26]	
Sahin et al., 2013 [27]	
Yilmaz et al., 2016 [28]	
Hayreh et al., 2002 [29]	
Chabanel et al., 1990 [61]	Blood viscosity
Arend et al., 1996 [62]	
Sofi et al., 2007 [63]	
Becatti et al., 2016 [64]	
Glacet-Bernard et al., 2011 [65]	
Donati et al., 2009 [66]	
Douat et al., 2007 [67]	
Heron et al., 2007 [73]	Abnormal erythroid differentiation
Wautier et al., 2011 [76]	Erythroid PS-mediated mechanism
Su et al., 2018 [78]	
